# Transition from routine arterial blood gas testing to indication-based testing in the intensive care unit: A sustainability in quality improvement initiative

**DOI:** 10.1016/j.fhj.2026.100528

**Published:** 2026-03-26

**Authors:** Richard Anderson, Debra Chalmers, Louise Trent

**Affiliations:** Intensive Care Unit, Hawke’s Bay Fallen Soldiers’ Memorial Hospital, Te Whatu Ora Te Matau a Māui Hawke’s Bay, Hastings, New Zealand

**Keywords:** Sustainability, Quality improvement, Arterial blood gas, Indication-based testing, Intensive care

## Abstract

Unnecessary routine blood testing is associated with patient harm, increased length of stay, increased cost, increased environmental harm and reduced nursing efficiency. Transition from routine arterial blood gas (ABG) testing to indication-based testing in one intensive care unit in New Zealand led to significant reduction in ABG testing and improved sustainable value without causing harm to patients. This was achieved through a Sustainability in Quality Improvement (SusQI) project involving a guideline, education programme, visual cues and audit and feedback. The impact has been sustained for 2 years post-intervention.

## Introduction

Unnecessary routine blood testing is an example of low-value care.^1^ It has minimal or no benefit and is associated with patient harm, increased length of stay, increased cost, increased environmental harm and reduced nursing efficiency.^2^ Studies estimate that 33–60% of arterial blood gas tests (ABGs) in intensive care units (ICU) are performed unnecessarily and that only 40% of ABGs in ICU result in a change in patient management.^2^, ^3^

Various interventions have demonstrated effective reductions in routine blood testing without deterioration in patient outcomes.^2^ For example, the ‘Choosing Wisely’ campaign is a global initiative aiming to reduce tests that are unnecessary and/or harmful. The campaign is supported by more than 80 medical specialist organisations across 30 countries. Indeed, the American Critical Care Societies Collaborative’s number one ‘Choosing Wisely’ recommendation is ‘*do not order diagnostic tests at regular intervals (such as every day), but rather in response to specific clinical questions’*.^4^ Additionally, several intensive care societies have developed sustainability recipe books to ensure that delivery of healthcare is considered in the context of environmental sustainability.^5^, ^6^ This is vitally important, given that healthcare accounts for 4–5% of global carbon emissions and ICUs are a significant contributor to this.^7^ It is estimated that low-value care makes up more than 30% of healthcare delivery and it is therefore an essential target for de-implementation as we aim to decarbonise the health sector.^8^

Despite this, the study ICU and many others have been practising ABG testing routinely after adjustments to organ support or at regular time intervals.^3^ The minimum frequency of ABG testing in the study ICU was previously determined by resident doctors, who would document how often bedside nurses should perform testing for each patient, with additional testing performed at nurses’ discretion. The study ICU decided to transition to an indication-based approach to ABG testing through a multi-faceted intervention. The objectives were to reduce the number of ABGs performed in ICU and therefore reduce harm to patients, reduce costs, reduce environmental harm and improve nursing efficiency. The Sustainability in Quality Improvement (SusQI) framework was applied to measure patient outcomes against a ‘triple bottom line’ of economic, environmental and social impacts.^9^

## Methods

### Context

The study ICU is a 13-bed regional ICU in New Zealand with 800–1,000 admissions per year. ABG testing is requested by medical or nursing staff and performed by bedside nurses using a point-of-care analyser.

### Intervention

An indication-based guideline for ABG testing was developed following a literature review and expert consensus from consultants and senior nurses in intensive care medicine. The guideline was subsequently refined using feedback from bedside nurses. The guideline was implemented in November 2023 in conjunction with the delivery of a departmental education programme to multidisciplinary staff in small groups by nurse educators and a resident doctor. Visual cues regarding the indication-based approach were placed at the point-of-care analyser. Audit and feedback took place after implementation.

The guideline recommended that ABGs should be performed on an individualised basis in response to clinical assessment or a specific clinical event, not as routine. Generic indications for ABG testing were described, as well as specific advice for patients undergoing invasive or non-invasive ventilation and patients returning to ICU from outside environments such as the operating theatre or radiology. Blood gas monitoring for patients with acute respiratory distress syndrome (ARDS) was guided by the medical team in line with current best practice. The education programme facilitated explanation and discussion of the guideline.

The New Zealand Health and Disability Ethics Committees (HDEC) confirmed that the study was out of scope and did not require HDEC review (reference: 2023OOS17874). The study was registered with the Te Whatu Ora Te Matau a Māui Hawke’s Bay Clinical Audit Department. Individual patient consent was not required.

### Measures

Data were collected for all patients admitted to ICU for 12 months before implementation of the guideline and for 23 months after implementation. Data were collected from electronic records. Data from patients whose ICU admission spanned the periods both before and after implementation of the guideline were excluded from the analysis.

Baseline data were collected regarding patient demographics, illness severity and urgency of admission. Patient outcome data were collected regarding ICU length of stay and mortality. ABG frequency data were collected and reported per patient-day on ICU.

Continuous variables were reported as mean (standard deviation) or median (interquartile range), as appropriate. Binary and categorical variables were reported as numbers (%).

*t*-tests, Mann–Whitney *U* tests and chi-squared tests were used to assess differences before and after implementation of the guideline, as appropriate. Multivariate linear and logistic regressions were used to assess the impact of the guideline on ICU length of stay and mortality respectively, corrected for age, sex, APACHE III score and urgency of admission. Multivariate regressions were also performed for a subgroup of patients who received respiratory support (invasive positive-pressure ventilation and/or non-invasive ventilation), assessing the same outcomes and corrected for the same variables. Statistical analyses were performed using DATAtab software.^10^

The SusQI framework was applied to ABG frequency data to estimate economic, environmental and social impacts.^9^ Cost per ABG locally, including analyser costs and consumables, was confirmed to be NZ$14.34 per test. CO_2_ equivalent emissions per ABG were estimated at 49 g based on a previous life cycle assessment study.^11^ An environmental agency tool was used to convert the carbon footprint into an equivalent number of miles driven by an average petrol car.^12^ Nursing time per ABG was estimated at 6.5 min based on previously published data.^13^ Blood volume drawn was estimated at 6 mL per sample, representing 1 mL sample volume and 5 mL discard volume as per local guidance.

Feedback was gathered from multidisciplinary staff following education sessions. Feedback themes and responses to feedback were recorded.

## Results

Baseline data of the ICU patient population pre-intervention and post-intervention were deemed not to be significantly different, described in Table 1. Data for eight patients were excluded because their admissions spanned the periods both before and after implementation of the guideline.

**Table 1:** Comparison of baseline data and outcomes pre- and post-intervention.

	Pre-intervention	Post-intervention	p-value
**Baseline data**			
ICU admissions	826	1,597	
Age – median years (IQR)	66.29 (51.85–75.7)	66.44 (52.74–77.11)	0.385
Sex, men	465 (56.30%)	908 (56.86%)	0.792
APACHE III score – median (IQR)	52 (37–69)	52 (38–69)	0.765
Emergency admission	755 (91.4%)	1,461 (91.48%)	0.947
Frailty score – median (IQR)	3 (2–4)	3 (2–4)	0.883
Inotropes/vasopressors	381 (46.13%)	745 (46.65%)	0.806
Invasive ventilation	194 (23.49%)	356 (22.29%)	0.506
Non-invasive ventilation	46 (5.57%)	74 (4.63%)	0.314
Renal replacement therapy	41 (4.96%)	67 (4.20%)	0.385
**Outcomes**			
ABGs per year	7,097	5,027	<0.001
ICU length of stay – median days (IQR)	1.97 (0.95–4.08)	2.07 (1.03–4.30)	0.054
ICU mortality	82 (9.93%)	171 (10.71%)	0.552

**Fig. 1:**
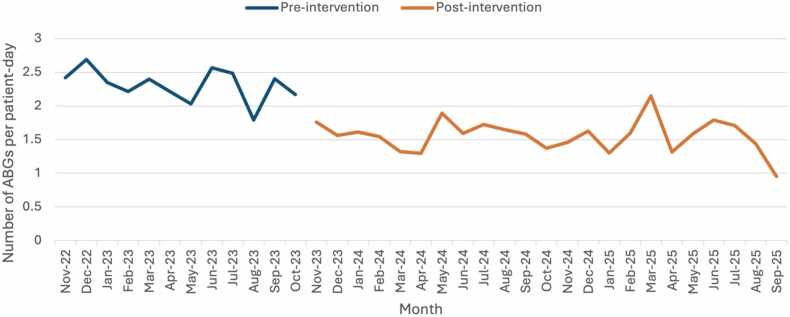
Monthly run chart demonstrating number of ABGs per patient-day pre- and post-intervention.

Transition to an indication-based approach to ABG testing led to an absolute reduction of 2,070 ABGs per year (7,097 per year pre-intervention vs 5,027 per year post-intervention) and a 32% reduction in ABGs per year corrected for bed occupancy (2.31 ABGs per patient-day pre-intervention vs 1.56 ABGs per patient-day post-intervention, p < 0.001). Fig. 1 shows a monthly run chart demonstrating number of ABGs per patient-day pre- and post-intervention.

Multivariate linear regression showed no significant difference in ICU length of stay pre- and post-intervention, when adjusted for age, sex, APACHE III score and urgency of admission (b = 0.38, 95% CI −0.15–0.91, p = 0.161). Multivariate logistic regression showed no significant difference in ICU mortality pre- and post-intervention, adjusted for the same variables (odds ratio 0.85, 95% CI 0.61–1.18, p = 0.321).

For a subgroup of patients receiving respiratory support in the form of invasive positive-pressure ventilation and/or non-invasive ventilation (n = 239 pre-intervention, n = 427 post-intervention), multivariate linear regression showed no significant difference in ICU length of stay pre- and post-intervention, when adjusted for age, sex, APACHE III score and urgency of admission (b = 1.08, 95% CI −0.67–2.82, p = 0.227). For the same subgroup of patients, multivariate logistic regression showed no significant difference in ICU mortality pre- and post-intervention, adjusted for the same variables (odds ratio 0.91, 95% CI 0.58–1.45, p = 0.702).

The sustainability implications of reduced testing are described in Table 2. There was an annual reduction in patient blood loss of 12,420 mL.

**Table 2:** Sustainability implications of reduced testing.

Sustainability factor	Estimated annual savings
Economic	NZ$29,683.80 (£13,654.55)
Environmental	101.43 kg CO_2_ equivalent emissions 258 miles driven equivalent emissions
Social	224 h of nursing time

Staff feedback themes and corresponding responses to staff feedback are described in Table 3.

**Table 3:** Staff feedback themes and responses.

Staff feedback themes	Responses to feedback
Will reduced testing be harmful?	Outcomes regularly audited and shared.
How did you decide when it is appropriate to perform blood gas testing?	Guideline developed following literature review and expert consensus from consultants and senior nurses in intensive care medicine. Final guideline approved by all consultants and senior nurses in department. Other staff able to provide feedback on the guideline post-implementation.
How will we be able to quickly decide about blood gas testing at the bedside?	Education programme for familiarisation. Guideline available at all bed spaces and the blood gas analyser for reference. Key message/visual cues: blood gases should not be performed routinely; they should only be performed if there is a clinical need. Queries can be raised with medical team.
Will the guideline be very restrictive?	Guideline intends to reduce unnecessary blood gas testing, but can always be overridden where a consultant intensivist deems it necessary.
How will impact be sustained in the long term?	Education programme including refresher case scenarios and induction training for new staff. Audit and feedback.
There is a problem with the guideline content or wording.	Guideline edited following feedback from multidisciplinary staff.
I’m not interested in sustainability.	Improving sustainability is a core responsibility of healthcare organisations, especially ICUs. In addition to environmental benefits, improved sustainability has implications for costs, patients and staff. Benefits regularly audited and shared.
This is a great idea and I’d like to get more involved.	Development of ICU green team. Opportunities to contribute to education programme.

## Discussion

Transition to an indication-based approach to ABG testing led to a statistically significant reduction in ABGs without a significant deterioration in length of stay or mortality. This was achieved through a multi-faceted intervention involving a guideline, education programme, visual cues and audit and feedback. Application of the SusQI framework demonstrated economic, environmental and social improvements as a result of reduction in ABG testing.^9^ The impact has been sustained for 2 years post-intervention. The findings are in keeping with previously published data and the duration of impact is longer than shown in some other studies.^2^ The work supports ‘Choosing Wisely’ recommendations to perform targeted testing rather than routine testing and demonstrates that broad sustainability value can be achieved without patient harm.^4^ This study is the first to report the impact of an ABG reduction intervention in a secondary-care general ICU.

We surmise that the success of this project is largely due to a multi-faceted approach and extensive involvement of multidisciplinary staff throughout the planning and implementation phases. Most ABG testing in ICU is initiated by nursing staff and it is therefore essential to involve nurses when transitioning to a new testing approach. Successful maintenance of the project was established through audit and feedback, including refresher case scenarios and induction training for new staff. Achieving engagement from all staff groups is thought to have supported implementation and longevity.

Limitations of the work include the before-and-after single-centre, non-blinded study design. In the absence of a control group, the number of ABGs performed pre- and post-intervention was corrected for bed-occupancy and recorded over annual cycles to account for contextual factors such as admission rates and seasonal variations in testing. While the reduction in ABG testing is assumed to be accounted for by reduction in ‘inappropriate’ testing, we did not measure compliance with the implemented guideline; however, the absence of excess harm to patients across the post-intervention period is reassuring. Additionally, previous studies have demonstrated that marked reduction in nursing-led testing is associated with small increases in doctor-led testing, which may be compensatory.^2^ Future compliance monitoring may be helpful in measuring and improving rationalisation of testing. Measures of harm were limited to mortality and length of stay. Further studies could include assessment of anaemia, need for blood transfusion, reintubation rates, patient satisfaction, harm associated with vascular access etc. This study demonstrated no significant additional harms for a subgroup of patients receiving respiratory support and further subgroup analyses should also be considered, for example related to specific reasons for admission. A large randomised controlled trial is likely to be required to detect changes in harm and to draw meaningful subgroup conclusions. Environmental and social impacts were estimated using data published from other countries and may not accurately reflect the impact in this context.^11^, ^13^ However, the geographic isolation of New Zealand means that any reduction in testing is likely to have additional environmental benefits above those seen in other contexts.

Implementation of similar initiatives across other ICUs or focused on other clinical tests is likely to demonstrate additional value.

## Conclusions

Transition to an indication-based approach to ABG testing in the ICU led to significant reduction in ABG testing and improved sustainable value without causing harm to patients. The impact has been sustained over the course of 2 years post-intervention.

## CRediT authorship contribution statement

**Louise Trent:** Writing – review & editing, Supervision, Methodology, Conceptualization. **Debra Chalmers:** Writing – review & editing, Supervision, Methodology, Conceptualization. **Richard Anderson:** Writing – review & editing, Writing – original draft, Methodology, Investigation, Formal analysis, Conceptualization.

## Funding

This study did not receive any specific grant from funding agencies in the public, commercial or not-for-profit sectors.

## Ethics approval and consent to participate

The New Zealand Health and Disability Ethics Committees (HDEC) confirmed the study was out of scope and did not require HDEC review (reference: 2023OOS17874). The study was registered with the Te Whatu Ora Te Matau a Māui Hawke’s Bay Clinical Audit Department. Individual patient consent was not required.

## Declaration of Competing Interest

The authors declare that they have no known competing financial interests or personal relationships that could have appeared to influence the work reported in this paper.

## Data Availability

The data that support the findings of this study are available from the corresponding author upon reasonable request.
