# Supplementary material for: Transition from routine arterial blood gas testing to indication-based testing in the intensive care unit: A sustainability in quality improvement initiative
Source: Future Healthc J. 2026 Mar 26;13(2):100528. doi: 10.1016/j.fhj.2026.100528 (PMC13157046; doi:10.1016/j.fhj.2026.100528)
Supplement: Supplementary file 1 — Supplementary material [file mmc1.pdf]

ICU Arterial Blood Gas Analysis Guideline

Purpose:

- To ensure arterial blood gas analysis (ABG) in ICU is performed only when appropriate.
- To reduce unnecessary ABG, harm to patients, unnecessary costs and unnecessary environmental harm.

General principles:

- There is no role for routine ABG.
- ABG should ideally only be performed when the findings will alter management.
- Some information gained from ABG can be obtained from other sources e.g. pulse oximetry.
- ABG is not required after every ventilator change.
- Arterial lines should be removed as soon as no longer clinically indicated.
- Unnecessary blood testing is associated with patient discomfort, anaemia, organ dysfunction, infection, requirement for red blood cell transfusion, increased length of stay, increased mortality, increased cost and environmental harm.

Indications for ABG testing

- Admission to ICU (if ABG not recently performed)
- Desaturation requiring increase in $FiO_2 \geq 0.1$
- Increased ventilator support
- Clinical evidence of deteriorating respiratory failure e.g. new/worsening tachypnoea, accessory muscle use, cyanosis
- Unreliable pulse oximetry – i.e. perfusion index (PI) <0.3 despite use of multiple probes/locations
- Cardio-respiratory arrest
- Haemodynamic instability – this may include, but is not limited to, rapidly changing vasopressor support or the use of multiple agents
- Clinical evidence of deteriorating intra-cranial pressure
- Suspected metabolic acid/base abnormality
- Indication to assess/monitor haemoglobin, lactate, electrolytes, co-oximetry etc
- Following electrolyte replacement only in patients with very low levels and/or ongoing losses (where laboratory electrolyte testing is not being performed)
- Conditions/treatments with specific management protocols e.g. diabetic ketoacidosis, continuous renal replacement therapy, insulin infusion
- To assess effect of intervention following previously abnormal ABG – follow-up plan for abnormal ABG results should always be discussed with medical team
- SMO request – specific patient groups may require increased frequency of monitoring e.g. ARDS patients. SMOs may request additional ABG based on clinical need.

Notes on ABG testing in specific circumstances:

Acute Respiratory Distress Syndrome (ARDS)

- There is a role for ABG in the management of ARDS. These patients will require frequent blood gas monitoring and manipulation of their ventilation. Frequency of testing in these patients is to be guided by the medical team.

Invasive ventilation

- Routine ABG is not indicated in mechanically ventilated patients unless there is clinical evidence of deterioration or the patient has ARDS (as above).
- **Change in ventilator mode**
  - ABG may be indicated following change in ventilator mode when associated with significant change in alveolar minute ventilation or clinical evidence of deterioration. Discuss with SMO if there is uncertainty surrounding need for ABG. Otherwise, repeat ABG should not routinely be performed.
- **Weaning**
  - Do not routinely perform ABG during ventilatory weaning unless there is clinical evidence of deterioration.
- **Planned Extubation**
  - ABG post-extubation is unnecessary unless there is clinical evidence of deterioration.

Non-invasive ventilation

- ABG should be performed prior to initiation of NIV.
- Patients with hypercapnic respiratory failure should have an ABG performed at 1-2 hours and 4-6 hours post-initiation of NIV.
- Non-hypercapnic patients on NIV can be monitored by pulse oximetry without the need for routine repeat ABG.
- Further ABG is indicated only if persistent acidosis or clinical evidence of deterioration.
- Patients on long-term (home) CPAP for stable respiratory disease do not require additional monitoring for this condition alone.

Patients returning to ICU from operating theatre/radiology etc

- If no clinical evidence of deterioration and no other indication for ABG (as per previous page), routine ABG is not indicated.

Note contents of invasive ventilation protocol regarding End-tidal CO_2 and $PaCO_2$ monitoring:

- *"A normal $PaCO_2$ is 35-45 mmHg. End-tidal CO_2 measures a mixture of alveolar and dead space gas, and is lower than arterial CO_2. The gap between the two measurements is small in healthy patients, but in the critically ill increases in dead space, in V/ Q mismatch and in the gas distribution within the lung results in a large, and variable end-tidal to arterial CO_2 difference. This makes meaningful estimates of arterial CO_2 from end-tidal impossible."*
- *"End-tidal CO_2 is however useful as evidence of subglottic placement of the endotracheal tube, and as an indicator of cardiac output, in cardiac, or near cardiac arrest. As such it is a useful monitor modality for intubated patients and is used routinely in ICU."*
- The above two statements do not preclude the appropriate use of ABG monitoring.
